# Literary Note

**Published:** 1898-11

**Authors:** 


### LITERARY NOTE.

THE *Kansas City Lancet* is the name of the medical journal heretofore known as *Langsdale's Lancet.* It is edited and published by Dr. John Punton, who is professor of nervous and mental diseases at the University Medical College, Kansas City, Mo. We think the change of name a wise one, as personal journal titles never appeal to the professional public. We wish the *Lancet* continued success under its new name and management.

